# Some Remarks on Alcohol and Gastro-enterostomy for the Cure of Chronic Alcoholism; with Report of Cases*This paper was read before the Bexar County Medical Society. It has been revised, the number of the operations brought up to date, and also number of successes and failures noted.

**Published:** 1914-02

**Authors:** J. W. Kenney

**Affiliations:** San Antonio, Texas


THE
### TEXAS MEDICAL JOURNAL.
Established July, 1885

F. E. DANIEL, M. D., - - - - Editor, Publisher and Proprietor

Published Monthly.—Subscription, $1.00 a Year.

VOL. XXIX. AUSTIN, FEBRUARY, 1914. No. 8

The publisher is not responsible for the views of the contributors.

### Original Articles.

For Texas Medical Journal.

### Some Remarks on Alcohol and Gastro-enterostomy for the Cure of Chronic Alcoholism; with Report of Cases.*

BY J. W. KENNEY, M. D., SAN ANTONIO, TEXAS.

The reason for resorting to gastro-enterostomy for the cure of the condition known as chronic alcoholism was based upon the conviction that the abnormal appetite for alcoholic spirits came primarily from a diseased condition of the stomach. This conviction was strengthened by the fact that pathological conditions similar to those existent in the inebriate's stomach are relieved by surgical measures, and the further fact that the drunkard in giving a history of his case usually points to his stomach as the inciting cause of his trouble.

Experience has proven to my mind and to those associated with me that gastro-enterostomy will relieve the uncontrollable desire of alcoholic drink as promptly as it will the pain and discomfort due to a gastric or duodenal ulcer.

Of the forty-two cases operated upon to date there have been two deaths and twelve relapses. One death was due to angina pectoris, from which the patient was a chronic sufferer. Two-thirds of the relapses occurred in patients known to be deranged mentally as well as physically. When the alcoholized subject has fatty degeneration of the brain and other organs of his body, he cannot be regenerated, and should be treated like other incurable insane patients are treated. The difficulty, however, of distin-

*This paper was read before the Bexar County Medical Society. It has been revised, the number of the operations brought up to date, and also number of successes and failures noted.

guishing between the insanity due to lesions of the central nervous system and that due to reflex causes will always act as a barrier to the surgeon in eliminating incurable cases. Often the most severe mental and nervous symptoms disappear in like manner and almost as quickly as the craving for liquor. It has, therefore, been my policy when in doubt to operate. The satisfaction of having cured one seemingly incurable case I believe justifies such a course.

The following case illustrates this point:

A bartender, aged about 35 years, had been a steady drinker for fifteen years. During the last two years he would have nervous spells, as he termed them, followed by convulsions. The convulsions occurred at irregular intervals several times monthly. The attacks became so frequent and severe that he was unable to attend to his duties as a bartender. He was operated upon July 23, 1910. He has not had a convulsion since the operation, and attends to his duties as a bartender without indulging in intoxicants, and his wife says: "He is just fine."

In order to arrive at a proper understanding of chronic alcoholism, one must familiarize himself with alcohol as a chemical; alcohol when taken inside the human body as well as the alcoholized body itself. As a chemical, alcohol is simply water, only that an atom of hydrogen has been replaced by a hydrocarbon radical. It owes its poisonous property to a dicarbon called ethyl. Alcohol is never imbibed alone, and ethyl cannot be found alone, but both enter more or less abundantly into popular beverages that are easily found and largely drunk, such as beer, wine, whisky, etc. Alcohol when taken inside the human body at first stimulates and then paralyzes; thus we find the alcoholic fresh from his glass quicker to see and hear than his abstaining brother, but after a few moments we find his sense of hearing and color perception to be not only below that of the non-drinker but below that of his normal self. The muscular system in like manner quickly responds to the effects of alcohol. The casual observer at some of our fashionable balls cannot help but notice the improvement in the dancer after his first visit to the punch bowl. After each drink this stimulation becomes more and more pronounced, until shortly the idiotic gyrations of his body cease, and we find our energetic dancer growing somnolent and flabby—he has become partially paralyzed. The mind and will-power are in like manner affected by the drinking of spirits. The talented speaker or writer is at first made more eloquent and fluent and is able to conceal his defects, but this is quickly followed by the infra-normal condition indicated by loquaciousness, inattentiveness and barrenness of thought. When we pass on to a consideration of the lower passions, we find an apparently slightly different picture. This difference, however, is only apparent. The small amount of inhibitive power normally wielded by the rational man over his animal nature is lost, thus seemingly allowing this stimulating stage to begin anew, when the paralysis of the inhibiting power is established. Inevitably, however, exhaustion follows.

Three things usually prompt the introduction of the drug into the body: (1) The taste, (2) its effect, (3) its foodal property. The first is of so little consequence that it need not be discussed; the second we have already considered, while the third has been demonstrated scientifically to be only a fancy, for the drug passes through the body unassimilated and unchanged, and never builds up or repairs tissue. There is even grave doubt that by checking the deposit of urea it stops nitrogenous decay and thus even remotely preserves life.

Upon entering the stomach, alcohol at first increases the flow of gastric juice, stimulates peristalsis and invites the taking of food, then destroys the power of the gastric juice by coagulating its pepsin, and stops the churning motion of the stomach by partially paralyzing the gastric branches of the pneumogastric nerve. The food is thus left to ferment and be regurgitated or to pass on down the digestive tract in a partially digested state, thus establishing a foci for auto-infection and its long chain of morbid phenomena. In the stomach and throughout its journey through the human system, alcohol displays a great affinity for water, thus depriving protoplasm of the water necessary to preserve it. This causes the drunkard to complain of thirst, which is a demand of the desiccated system for water. This demand is usually met by the addition of more alcohol. Constant repetition of this procedure results not only in pickling our protoplasm, but in a degeneration of all the organs and tissues of the body. The stomach is the first organ of the body to present evidence of the destructive power of alcohol. In this organ, we find two conditions existent: (1) A dilated stomach with thin walls and presenting a mucous membrane with numerous hyperemic ulcerated and disintegrated spots. (2) A contracted stomach with thick tough walls and a lining membrane of practically cicatricial tissue. To relieve the ill-effects of such conditions when produced by any other agent than alcohol, a gastro-enterostomy or some similar operation must be performed. In my endeavor to apply this same principle for the relief of the alcoholic stomach, I have found a posterior gastro-jejunostomy most quickly and easily performed, and practically without danger to the patient's life. By the time the patient is able to leave the hospital, the desire for alcoholic spirits will have, in a majority of cases, disappeared and his mental and nervous condition will show decided improvement. These points are well illustrated in the case of a young civil engineer sent to me from a distant State by his father. Upon his arrival, he was found to be in such poor condition, both physically and mentally, that I refused to operate upon him until he had recuperated by spending three months roughing it on a ranch. Upon his return, the operation was performed and his condition at present cannot be better described than to quote one passage from a letter received from him under date of February 20, 1911. He states: "As you know, I have taken so-called cures, which never gave me relief from the desire to drink. With the operation, I am for the first time in twenty years free from my only trouble. Am getting fat and am perfectly healthy, and my nervous system is completely restored to what it should be."

The technique of the operation as performed by me does not differ in any material respect from the gastro-jejunal anastomosis made for the relief of other gastric disorders. The most dependent part of the body of the stomach is usually selected and is attached to the jejunum at a point fifteen or twenty inches from the duodenum. Catgut sutures are used entirely, and pads of gauze wrung out of warm vaseline are used to cover exposed omentum, bowels, etc. The use of the latter, I believe, adds materially to the comfort of the patient during convalescence by preventing any irritation of the bowels during the operation, and, consequently less pain and fewer adhesions afterwards.

